# Autonomy & advocacy in planning for a medical emergency: Adults with a learning disability and family carers’ experiences and perceptions of the Recommended Summary Plan for Emergency Care and Treatment (ReSPECT) process

**DOI:** 10.3310/nihropenres.13555.2

**Published:** 2024-10-10

**Authors:** Amy M. Russell, Jacqui M Lovell, Jenny Harlock, Frances Griffiths, Anne Slowther

**Affiliations:** 1 Leeds Institute of Health Sciences, University of Leeds, Leeds, England, LS2 9NL, UK; 2 School of Psychology, Liverpool John Moores University, Liverpool, England, L3 3AF, UK; 3 Warwick Medical School, University of Warwick, Coventry, England, CV4 7AL, UK

**Keywords:** ReSPECT, emergency care, Emergency Care Treatment Plans, advance care planning, learning disability, carers, advocacy, autonomy

## Abstract

**Background:**

The Recommended Summary Plan for Emergency Care and Treatment (ReSPECT) is designed to facilitate meaningful discussions between healthcare professionals, patients, and their family about preferences for treatment in future medical emergencies. People with a learning disability may face particular barriers in completing health care plans and receiving emergency treatment, however little is known about their preferences towards emergency care treatment planning. This study explores the views of people with a learning disability, and family carers about ReSPECT.

**Methods:**

A reference group of 5 people with a learning disability contributed to the design of the workshops and evaluation of outputs. Online, arts-based interactive workshops were held with 2 groups of 6 people with a learning disability to explore how they felt about emergency care treatment planning, and to co-produce materials to support ReSPECT conversations. Carers of people with a learning disability participated in focus groups or interviews. Data from workshops, focus groups and interviews were analysed thematically.

**Results:**

Themes were; Getting the Process Right, Lack of trust a barrier to ReSPECT planning, and Person-Centred Care. All groups supported the ReSPECT process feeling that ReSPECT plans could support person-centred care, enhancing the autonomy of a person with a learning disability and supporting the advocacy of carers. However, drawing on their previous experiences of the health care system some expressed doubt that their wishes would be carried out. Suggestions were made for improving the ReSPECT process and used to develop resources to support ReSPECT planning.

**Conclusions:**

Emergency care planning and ReSPECT are viewed positively by people with a learning disability and family carers. To ensure this works well for people with a learning disability attention should be given to reasonable, personalised adjustments to support their participation in planning conversations. There is a wider challenge of fostering trust in the health care system.

## Introduction

Emergency Care Treatment Plans (ECTPS), sometimes called Treatment Escalation Plans (TEPs), have been developed as a mechanism for making considered recommendations about future treatment in the event of the person developing a life-threatening emergency or acute deterioration in an existing illness when they are unable to make a decision for themselves. Treatment recommendations, including recommendations about cardiopulmonary resuscitation (CPR), are made by a health professional in discussion with the person or their family, reflect the person’s preferences and values, and are intended to guide future treating clinicians ^1^. ECTPs are distinct from advance care plans (ACPs), where the focus is on end-of-life care preferences and extends beyond treatment choices ^2^. In the UK, the most widely used ECTP is the Recommended Summary Plan for Emergency Care and Treatment (ReSPECT). Developed in 2016 by the Resuscitation Council UK (RCUK) in response to concerns about stand-alone Do Not Attempt Cardiopulmonary Resuscitation (DNACPR) forms, the aim was to provide a national standardised approach to support conversations about goals of care and facilitate person centred decision-making in emergency situations ^1^.

People living with a learning disability in the UK are under served by healthcare services ^3^. Pre COVID the Learning Disability Mortality Review found that many deaths of people with a learning disability were avoidable ^4^. This population has higher rates of long-term health conditions and are more likely to be admitted to hospital as an emergency ^5^. During COVID-19, the rate of excess deaths for people with a learning disability in 2021 was more than two times higher than the general population ^6^.

People with a learning disability, have often been overlooked in advance care planning. Yet they may disproportionately benefit from advance planning as their needs and wishes are often not met in acute situations ^7, 8^. The 2013 Confidential Inquiry into premature deaths of people with learning disabilities (CIPOLD) exposed significant failings in healthcare for people with a learning disability, making multiple recommendations including reasonable adjustments to care processes and changes to advance care planning and how people’s care is managed at end of life and in an emergency ^7, 9^. In 2018 NICE recommended emergency care planning be part of health and social care planning for people with a learning disability but did not detail how this should be carried out or recorded ^10^.

Research from high income countries with a variety of healthcare systems suggests that the involvement of people with a learning disability in decision-making about planning for their care and treatment in an emergency is minimal ^11, 12^. A more recent systematic review, including studies from the UK, reinforced these findings ^13^. Reasons include, but are not limited to, communication challenges, lack of education/training on caring for individuals with a learning disability among health professionals, assumptions that the individual lacks capacity to consent merely due to the presence of a learning disability, assumptions that people may get too upset or not wish to discuss negative outcomes ^14^ and fear of legal consequences if medical professionals are accused of not providing enough care ^15, 16^. The lack of involvement in decision-making can have adverse consequences including lack of comfort or increased distress at end-of-life, unwanted hospitalisations, unnecessary life-extending treatments, and foregoing personal wishes for care and treatment ^16^. Furthermore, scholars have expressed concerns that without detailed emergency care plans people with learning disabilities have been denied active care ^17^.

Studies that have explored advance care planning with people with a learning disability often focus on the views of relatives or health professionals ^18^. Those studies that have explored ACP with people with a learning disability have found key considerations in planning to be; pace, appropriate support, and adaptations to the process based upon individual needs ^11, 19^. Some studies have highlighted concerns over clinician preference for proxy decision making, when family members make decisions on behalf of a person with a learning disability, thus undermining an individual’s autonomy ^20, 21^. Where no ACP is in place family members can be called upon to make tough decisions in emergency contexts, sometimes without ever having discussed with the individual their preferences for emergency care ^8, 12, 19, 22^.

Studies including the perspectives of people with a learning disability benefit from the involvement of people with a learning disability to shape the study design and process ^23^. Inclusive research aims to move research from being about people with a learning disability to research carried out with them ^24^. This change allows for people who had previously been the subjects of research to be active agents in the design and delivery of research. The benefits of this approach are numerous; research can be more meaningful to a population, representative of their perspectives and impactful as it has already engaged with context and lived experience of the subjects of that research ^25^. This study is part of a growing body of inclusive research in the context of health.

We report on a study that explored the views and experiences of people with a learning disability and family carers on the concept and practice of emergency care treatment planning in general and ReSPECT in particular. We worked with people with a learning disability to produce materials to support people with a learning disability and health and social care professionals working with them, to prepare for ReSPECT planning conversations. This study was part of a programme of research evaluating the use of ReSPECT in a primary and community care setting ^26^.

## Methods

### Overview of study

The study had two elements; a series of workshops with people with a learning disability and focus groups or interviews with family-carers of people with a learning disability. CHANGE allocated two different members of staff, one of whom has lived experience of a learning disability, to recruit to the project, facilitate the workshops, and support the reference group for the workshops.

We ran five workshops with each of two groups of six participants who had a learning disability to explore their views and perceptions of emergency care treatment planning and how it might work for them, and to co-produce support materials for healthcare professionals to use in emergency care treatment planning.

We convened a Reference Group of people with lived experience of a learning disability to co-design the workshops with the study team, contribute to analysis of the workshop findings, and review the co-produced resources. The reference group was also invited to participate in an autoethnographic evaluation of their experience as part of the study. Thus, reference group members occupied a dual position as PPI members of the study team and study participants in the autoethnography. We have reported the autoethnography elsewhere ^27^.

Family carers of people with a learning disability were invited to take part in an online focus group or individual semi-structured interview to share their experiences and perspectives regarding emergency care treatment planning for both themselves and the person they cared for. Focus groups were managed by the core study team.

### Patient and public involvement

We had identified that neither the voices of people with a learning disability nor their carers were being heard in the wider research project. We decided to create an additional work package that would focus on the experiences of people with a learning disability and their carers. We partnered with CHANGE a human rights charity led by people with a learning disability based in Leeds, and part of the Advonet Group ^28^. CHANGE operates a co-working model, employing people with learning disabilities to co-run projects, co-deliver training and co-create accessible information. Two CHANGE members worked with the study team to develop the study design and, later, the participant facing documentation. We convened a Reference Group of people with lived experience of a learning disability to co-design the workshops with the study team, contribute to analysis of the workshop findings, and review the co-produced resources. A further two CHANGE members, one with lived experience of a learning disability, worked with the study research fellow (JL) to facilitate reference group meetings and study workshops.

Reference Group members were recruited through CHANGE’s national networks of learning disability organisations. An email with relevant study information was circulated and interested participants were asked to contact CHANGE directly. CHANGE provided further study information, checked eligibility and capacity to participate in the Reference Group, and that prospective participants would be able to attend meetings in person at CHANGE. Further easy-read information and a consent form was then provided to eligible, interested participants, and a second contact arranged to answer further questions and take consent. Five members were originally recruited, however the reference group initially struggled to retain members due to their own personal circumstances therefore CHANGE entered a second round of recruitment following the same procedures as above. Thus, the initial preparatory meetings for the first workshop were held with the remaining participant and the facilitators from CHANGE. Three additional reference group members were then recruited by CHANGE following the protocol. The four final reference group members attended all remaining reference group meetings. Reference group, members received a shopping voucher after each meeting. 

Reference group members co-designed the activities in the workshops, reviewed the findings from the workshops and the carer focus groups and collated them to create a presentation for the stakeholder conference. Reference group members presented the work package findings at the stakeholder conference with the lead researcher. A report of the stakeholder conference and its recommendations was made accessible and presented to the reference group to inform their review and refining of the resources prior to final versions being produced.

One member of the workshops was working with an NHS trust on a Dying Matters event. They invited AR to present with them at the event. Because of their involvement and interest in advance care planning they were invited to attend the reference group meeting to review and feedback on the resources developed. 

 A summary of relevant chapter of the final project report to funders was created in an accessible format and presented to the reference group for feedback. This was revised following their comments.

The reference group conducted a collaborative autoethnography of their experiences of the project. We are planning to co-author a paper with the Reference Group on their experiences of working on the project so these data do not feature here. CHANGE will support the dissemination of findings though their networks.

### Recruitment

To recruit to the workshops CHANGE disseminated relevant study information to national networks of learning disability carer and self-advocacy organisations. Individuals interested in participating in the workshops contacted the project lead at CHANGE by telephone or email. The CHANGE project lead responded to check eligibility (aged 18 years or over, capacity to consent to participate in co-production workshops, resident in England, internet access and able to use online platforms either themselves or with a supporter). If eligible, the CHANGE project lead explained more about the project and answered any questions. Further written information was provided and a second contact arranged to seek consent to participate in the workshop series. Prior to obtaining consent the CHANGE project lead checked that the person had read the information sheet, had it explained, that any questions had been answered, and the person understood what was involved in workshop participation. Verbal consent was then obtained and documented. At the start of each workshop the CHANGE project lead re-checked consent to participate and to record the meeting with all participants. Workshop participants received a shopping voucher for their time after they attended each workshop.

We sought expressions of interest for focus group participation from family carers of people with a learning disability through CHANGE’s contacts of learning disability and learning disability carers’ support organisations, and through online carers’ support forums and carers’ groups identified through social media platforms and internet searches. Potential participants contacted the study team directly and were provided with further information and offered a range of dates for planned focus groups. If they could not join the planned focus groups we offered an individual interview. All focus groups/interviews were held online, and participants were given a unique link to a Zoom™ meeting for the relevant focus group/interview. Prior to the focus group/interview, participants were sent a blank copy of the ReSPECT plan for reference, and a consent form. Prior to commencement of the focus group/interview the researcher went through the consent form with the participant and recorded their responses to each section. Participants received a shopping voucher as a thank you for participation.

The study was approved by London South East Research Ethics Committee (REC reference 21/LO/0455 date of approval 17.06.2021).

### Workshop development and role of the reference group

The Reference Group met initially to familiarise themselves with the research, establish ways of working, and to plan the first workshop. They then met in-between workshops to receive feedback from the previous workshop(s) and plan the next. A total of eight meetings took place, with the final one dedicated to reviewing resources produced by workshop participants to support ReSPECT planning, and to reflect critically on the overall process and learning. A researcher (JL), along with the designated CHANGE staff member, attended both the reference group and workshops throughout to provide feedback from the workshops to the Reference Group members, link issues raised, and make connections with wider study findings. With consent, reference group meetings were audio recorded to supplement fieldnotes made by the researcher. A more detailed description of the iterative process between reference group and workshops is reported elsewhere ^27^.

***Workshops with people with a learning disability.*** Five workshops were held each with six participants. Workshops were held online and facilitated by the researcher (JL) and two members of the CHANGE team (ID and SW). The second series of workshops began after the third workshop in series one so lessons learned from series one would inform series two. Workshop one involved providing information about emergency care treatment planning to participants and exploring their experiences of accessing primary care, familiarisation with the ReSPECT process and form, using story sheets (see supplementary material for examples of workshop support materials). Workshops two to four explored participants views of the ReSPECT process in more depth, including their suggestions for what were important prerequisites to support people with a learning disability in preparing for a ReSPECT planning conversation. Workshop five summarised what had been learned in the workshops and provided an opportunity for reflection and any additional comments or suggestions for resources to support people with a learning disability. Workshops were recorded with consent and a summary of each workshop was drafted by the researcher (JL) using field notes and the recording.

***Focus groups and interviews with carers of people with a learning disability.*** A topic guide was developed by AR and JH and refined by a family carer and chief executive officer of a charity supporting people with learning disabilities (Special Needs Outreach Project (SNOOP) ^29^. The topic guide prompted participants to consider potential benefits and disadvantages of emergency care treatment planning for people with a learning disability and their carers; how an emergency care treatment planning process could be implemented with people with a learning disability, the role of carers in the process and a carer’s own emergency care planning in light of their caring role. Focus groups were facilitated by up to three researchers (JL, a critical community psychologist and expert in participatory research methods; AR, an applied health researcher and expert in health inequalities and learning disability, and JH, a social and policy scientist and qualitative researcher). All individual interviews were carried out by JL. All focus groups and interviews were recorded and transcribed.

Focus groups lasted between 38 and 76 minutes and interviews between 45 and 62 minutes.

### Analysis and developing resources

Workshop summaries and focus group/interview transcripts were analysed using thematic analysis ^30^. JL and AR read a sample of workshop summaries initially coding deductively for issues relating to information, process, and support for engaging with the ReSPECT process (which fed into development of resources). The summaries were then re-read and coded inductively to capture participants views on the meaning of emergency care treatment planning for people with a learning disability. JL then coded all summaries and identified candidate themes which were then discussed and refined with AR and presented to the Reference Group. Focus group/interview transcripts were coded initially using the topic guide areas as a thematic framework. The themes identified from the workshop data were process, autonomy, and trust; and those from the focus groups with relative-carers were process, lack of trust in the system, and carers as advocates. Given the resonance noted between the themes from each data set AS then read the sections of summaries coded under corresponding themes for each data set (process, trust/lack of trust, and autonomy/advocacy), and identified themes that captured meaning across both data sets. These were discussed and refined with AR.

To develop resources to support people with a learning disability preparing for ReSPECT planning, researchers (JL) and (AR) reviewed all workshop summaries to identify key messages and suggestions around information, process, and support for engaging with the ReSPECT process. JL presented to the reference group the workshop findings and a summary of a stakeholder meeting held as part of the wider ReSPECT project and attended by members of the reference group. The group provided further feedback on the type and content of the resources, then two reference group members, one workshop participant who had experience of work on advance care planning, together with JL and the CHANGE team, prepared the content and format of the recommended outputs. These were then submitted to CHANGE to convert into Easy Read format. The full reference group reviewed draft versions and recommended minor modifications. Resources can be found here ( https://warwick.ac.uk/fac/sci/med/research/hscience/sssh/research/respect/resources/ or here https://www.resus.org.uk/respect/respect-resources see Resources to Support those with learning disabilities).

## Results

Twelve people with a learning disability took part in the workshops. Five family carers took part in a focus group (and seven took part in six interviews (one parent couple took part in a joint interview). Eight of the twelve carers were aged over 60 years and ten were female.

Three themes were identified. Getting the process right, Lack of trust as a barrier to ReSPECT planning, and Person-centred care: Autonomy & Advocacy.

### Getting the process right

Overall participants with a learning disability and family carers were supportive of ReSPECT planning as a way of ensuring care and treatment in an emergency was tailored to the needs and preferences of the person whose plan it was.

Carers said that developing a ReSPECT plan for someone with a learning disability required careful thought about the needs of the person and who should be involved in the conversation. They all agreed that any ReSPECT conversation must include them as the primary carer and most felt that they should lead the conversation.

“I think the parents gotta lead on that…because we know [name of person] better than anyone else”
                                                                                  [Parent carer Interview 1]

However they also emphasised the importance of including members of the multidisciplinary team providing care for the person and members of their family. One participant expressed that they would want support from someone who understood the process so they would feel confident in completing the plan for the person they cared for.

“So, I think in the room with me would have to be somebody who could actually break down this form in really simple lay terms that I knew that whatever I was putting on there was as I understood 150%. There's no scope for ambiguity, or there's no scope for me to get anything wrong.”
                                                                                  [Parent carer focus group 2]

Workshop participants also emphasised the importance of getting the process right so that a person with a learning disability was supported and empowered to have their voice heard and reflected in their plan. Key to this was support to prepare for a ReSPECT conversation and support following completion of the plan to enable the person to reflect and consolidate their understanding of their plan. Having the right person to support them through this process was important. Reflecting on their creative representation of their needs for ReSPECT planning one participant explained;

“...I did a picture of a hand but you've gotta have the right support, like people or person to support you. So basically, it's like you've gotta have the right hand ...”
                                                                                  [Workshop group two]

Support was requested before, during and after the appointment for the ReSPECT conversation. Participants were concerned that the discussion may distress them and they would need to debrief afterwards.

*“just thinking about the emergency coming on. Well, if you’re a person with the learning disability, that in itself can be scary.”* [Workshop Group 1]
*“…after the appointment, I'd like to go over everything and make sure I've got it all straight and asked everything I wanted to.”* [Workshop Group 1]

They emphasised the need for time, both for preparation and during appointments for the discussion of ReSPECT.

*“I would need time to process the information what was given to me. Yeah, so I repeat myself and say I don't understand what is being said. ...they say it really fast and they don't give me time yeah. Then I would need maybe to go back again and say, look, can you repeat that please? Cause I didn't understand it.”* [Workshop Group 2]

Several participants said they did not get double appointments with their GP despite this being a recommended reasonable adjustment. The importance of having sufficient time to ensure that the plan properly reflected the person’s needs and preferences was a consistent finding across all our data.

“ *I'm a very reflective person and, and if I'm doing it for somebody I'm caring for, I'm going to be even more reflective, so I want to go away and I want to look at the document. I want to digest it”* [Parent-carer Focus group 2]

All carers felt it was important to do this process early and review it regularly.

Several participants with a learning disability expressed the need for reasonable adjustments to the conversation with a clinician which apply more broadly to health appointments, for example, quiet waiting spaces, a clinician familiar to them, longer appointments, easy read information, alternative formats including videos to support understanding the process and a supporter present.

Workshop participants also wanted the language on the ReSPECT form to be understandable for them as the current version of the form needed further explanation. They emphasised accessible language explanations should be on the form and not in a separate document that may or may not be given to them.

“when you're seeing the DNACPR… I was thinking what does that mean? So, I thought like if they had it in brackets, what it meant” [Workshop Group 1]
“especially … if you can't even understand or read it, how on earth are you going to be able to know what [CPR] means?” [Workshop Group 1]

### Lack of trust as a barrier to ReSPECT planning

Several workshop participants described feeling that their learning disability distorted assessments of their other health care problems and needs during clinical appointments, and they thought this may also be the case in completing a ReSPECT plan. Describing their pictorial narrative of a ReSPECT journey, one participant identified as a key challenge, health care professionals not listening or understanding the needs of someone with a learning disability.

“mine is ... is being afraid to talk to a doctor and a doctor not listening to someone who's got a learning disability and they can’t understand someone with a learning disability ... Well where you gotta tell...you gotta tell the doctor you got a learning disability and they don't listen to you as well. That, that he could be a big hurdle to get over.”
                                                                                  [Workshop group 2]

Family carer participants also referred to previous experience of negotiating with the health care system when seeking treatment for the person with a learning disability they cared for, leading to some doubt about how the ReSPECT process would work in practice. They expressed uncertainty about who would have the form and whether it would be read and acted on in an emergency.

“Not very, not very, because again, it comes back to where who's got the form. Can they find it when you need it? Is it up to date? Does the person who access the form actually have they, have they read it? Do they understand it? Yeah, ... I think things are only will only happen if I do it myself”
                                                                                  [Parent carer Interview 2]

Both carers and people with a learning disability referred to their experiences with Hospital Passports (another patient/carer held record), explaining that patient held forms are often not requested, nor read when presented.

Carers also worried that even if a plan was available, recommendations would not be followed if they were not present to reinforce them. For example, a parent carer, while expressing a lack of confidence in general about ReSPECT plans being followed, noted that her son’s was likely to be followed simply because she would be there to reinforce it, which would not happen without that advocacy.

“that's another one entirely, isn't it, I would have more confidence with my son’s because my son would have me behind it and I would be driving it and making sure it was present, that it was found that it was read, that it was listened to, that it was discussed and dissected and acted upon. So that's that me, yeah, somebody by themselves, yeah not, not confident.”
                                                                                  [Parent carer Interview 3]

However, some carers did have confidence that ReSPECT recommendations would be followed. Others accepted that sometimes external factors impacted on the ability/likelihood of health care professionals accessing or following the recommendations.

“I do have, no, I do have confidence, I do. I think people are in the job for the reasons they should be in the job.”
                                                                                  [Parent carer Interview 5]
“The thing is, you know, the real, the, the reality is that at the time of the crisis, and we don’t know whether there's a, a strike with the with the ambulances or whether that person’s stressed and she’s already got three people with passports and she can’t possibly read them all.”
                                                                                  [Parent carer interview 6]

Yet for many repeat experiences of discrimination toward their child with a learning disability, exacerbated by the pandemic, meant they had little trust in the current system’s level of care for people with a learning disability. Several parent carers reported cases of healthcare professionals making assumptions about their adult child’s quality of life. As one carer described,

“we saw it with COVID and it's terrifying. I think that's the thing that scares me more than anything is that it's, you know, I stand up there clapping, and they deserve to be clapped as nurses you know. But at the same time, I was thinking that ‘these are the people who won't treat my son.’ If he goes into hospital, these are the people who've decided he is not worthy of the ventilator”
         [Parent carer Interview 2]

### Person centred care: autonomy and advocacy

For our workshop participants the ReSPECT process provided an opportunity for them to be able to say what they wanted in terms of their future treatment and care. It provided recognition and respect for them as a person and reminded health care professionals in a future emergency that this person’s wishes should be respected.

“I’m in my right mind to make sure what I want is in place. I have a disability but know what I want yeah.”
                                                                                  [Workshop group 1]
“I wanna put down what I want to happen, how I want people to treat me or speak to me…”
                                                                                  [Workshop group 2]

However, based on their previous experiences they were concerned about the capacity assessment within the ReSPECT planning process and whether assumptions would be made that they lacked capacity. This would undermine their autonomy.

“[the form] doesn’t say how anybody decides that person’s capacity”.
                                                                                  [Workshop Group 1]

They felt strongly that any capacity assessment should be done transparently and recorded clearly, so it was apparent to someone reading the form that the values expressed were their own.

“Absolutely there should be an explanation for what my capacity is in there because, yeah, it's important. This is your form you should be able to know every single thing that’s on it.”
                                                                                  [Workshop group 1]

Family carers also thought a ReSPECT form could help clinicians responsible for making treatment decisions in an emergency when the carer may not be present to advocate for that person. This was seen as particularly important if the person with a learning disability had complex needs that the clinician may be unaware of.

“And also with [person with a learning disability’s name] you can’t learn anything straightaway because he might start gabbling and you think, “Oh, he’s just...is he...” you don’t expect him to be, have the, the disabilities that he does have, you know, so...yeah, it’s got to be a help. It’s got to be a help, having some more information.”
                                                                                  [Parent-carer interview 6]

Carers also thought the benefit of a ReSPECT plan was in providing a holistic picture of what was important to the person with a learning disability for the clinicians treating them in an emergency, so that treatment decisions were tailored for them and not based on assumptions.

“I would like to think that this form would be useful for them in making sure that a considered discussion has taken place with the clinicians prior to any emergency situation which clearly states the intentions of, of that family member because what we don't want is some high handed medic taking over and thinking they know what is in the best interest for that particular patient”
         [Parent carer interview 3]

However, in most circumstances carers saw themselves as representing and advocating for their family member in person, as they had always done. The ReSPECT plan was seen as a safeguard if they were not available to speak for the person with a learning disability but that it was unable to evolve to meet changing needs and circumstances. For one participant their advocacy role reduced the relevance of a ReSPECT plan.

“… I personally do not see the need for one for [son’s name] because I am his voice. I am his advocate and I'm always the person that is involved in every aspect of his care.”
                                                                                  [Parent carer interview 3]

For some carers, their focus on ensuring best care for their relative with a learning disability framed how they considered their own ReSPECT plan, their preferences regarding future treatment being driven by the impact they would have on the future care of the person they cared for.

“Erm, I think ... my daughter has a very difficult road ahead with [son’s name] looking after [son’s name] when I am either not here or completely unable to assist and for me, [son’s name] is the priority. Therefore, I don't want anything for me that's going to make her feel, ‘oh Lord, now I've got to look after mum too,’ so I'm not entirely sure I would want something that's made, enabled me to survive, but disabled me entirely”
                                                                                  [Parent carer interview 1]

### Developing resources to support people with a learning disability in ReSPECT planning

From our analysis and discussion with the reference group we developed an Easy Read guide for health and social care professionals to use with someone with a learning disability to explain RESPECT, identify support needs for the person during the process, and explore what the person wanted to include in the plan. The resources included worksheets that could be used for the person to set out their needs and plan what they wanted to say. These were based on resources used and refined in the workshops. Reference group members felt strongly that storytelling and creative methods were a way to support a person with a learning disability to articulate what was important to them. They emphasised the need to focus on preparation, having the right people present and practical planning, like transport to a ReSPECT conversation. They felt poor access to services could derail the ReSPECT process before it began. All resources developed are available at RCUK and on the study website.

## Discussion

Our participants were mainly positive about the ReSPECT process. They felt it supported person-centred care, created an opportunity for a person wishes to be heard and recorded, and provided a backup to the advocacy of carers. However, enthusiasm was tempered by doubts about the ReSPECT process given their previous negative experiences of disability discrimination in healthcare. Suggestions were made to reasonably adjust the process of making a plan to support people with a learning disability to be involved and to ensure plans contributed to improved person-centred care in an emergency.

Family carers spoke about ReSPECT as a safety net if an emergency happened and they were not available. However, given their long history of fighting for the rights of their child or sibling, often in response to healthcare professionals' behaviour and attitudes they felt their role as advocate remained vital. ReSPECT was seen as secondary to their personal communication with the clinicians making treatment decisions for the person they cared for. Studies with older adult carers in settings other than health such as housing have also found resistance to engaging in future planning conversations ^31, 32^. Carers may struggle to delegate responsibility for care to any person or process after years of acting as the main advocate for their adult child. However, findings suggest that, if done sensitively, carers can welcome the reassurance such planning provides ^33^.

Family carer participants’ previous experiences of discrimination within the health care system led to a lack of confidence that a ReSPECT plan would be accessed and followed, and hence a lack of trust in the process. Their negative experiences of health care resonate with previously published accounts including the Confidential Inquiry into Premature deaths of people with a Learning Disability (CIPOLD), the subsequent LeDeR reports and the mistakes in care that necessitated the creation of the Oliver McGowan mandatory training ^4, 7, 34– 36^. Concerns about inappropriate use of DNACPR recommendations including lack of consultation, support and accessible information for people with a learning disability were highlighted in the Care Quality Commission’s review of DNACPR decision-making during the COVID 19 pandemic ^37^. Analogies to the Hospital Passport scheme by people with a learning disability and family carer participants raised concerns that a patient held plan would not be asked for and, if provided, would not be read and adhered to, in an emergency. McCormick *et al*. found similar concerns in their interview study of the use of a Health and Social Care Passport in Northern Ireland ^38^. In addition, participants with a learning disability expressed concern that assumptions would be made about their capacity, or lack thereof, to participate in the ReSPECT planning process. Similar concerns about assumptions around capacity, and poor compliance with capacity assessment requirements in health care decision-making for people with a learning disability were found in a 2019 systematic review of studies evaluating how health and social care professionals assess mental capacity ^39^.

Workshop participants with lived experience of a learning disability focused primarily on the reasonable adjustments that were needed to support the conversation to complete a ReSPECT plan. They specified generic adjustments which they were not currently getting but that would be reasonable to comply with the Accessible Information Standard ( https://www.england.nhs.uk/about/equality/equality-hub/patient-equalities-programme/equality-frameworks-and-information-standards/accessibleinfo/) and would be good practice in any primary care appointment, for example Easy Read/alternative format, double appointments, accessible communication from practices. In addition, they expressed a range of personal preferences for communication and adaptations to the ReSPECT process that would best be identified though a discussion with the person and a supporter. Guidelines for the development of reasonable adjustments for people with a learning disability to support equity of access to health care have been produced in other specific areas such as diabetes care ^40^ (see for example How to make reasonable adjustments to diabetes care for adults with a learning disability, www.diabetes.org.uk/learning-disability).

In addition to appropriate information delivery our participants with lived experience of learning disability emphasised the importance of having the right person to support them at all stages of the process, and the time to prepare for a conversation, have the conversation, and reflect on the conversation afterwards. Family carers also emphasised the importance of having the right people involved in the conversation, and that conversations may be complex and require several discussions before a plan could be agreed. These findings highlight the importance of wider relationships in the care of a person with a learning disability, other than the relationship between patient and health care professional. This study is significant in that the primary focus was the thoughts, experiences and wishes of individuals with a learning disability, and family carers of people with a learning disability on anticipatory care planning. While the literature on emergency care treatment planning is limited there is a wide literature on Advance Care Planning (ACP). However, in a systematic review of ACP for people with a learning disability in palliative care (14 studies included), Voss et al found no studies which considered the views of individuals with a learning disability, only studies with healthcare professionals and relatives ^18^. In addition, they found no studies that considered the process of advance care planning, with studies mainly focussing on one element, that of end of life decision-making and/or DNACPR. This suggests that anticipatory care planning for people with a learning disability is rarely considered by both practitioners and researchers.

Our intention was always to co-design the workshops and the outputs of the project with people with a learning disability. This had been team members’ previous experience of research ^41, 42^ and supported our commitment to inclusive research. A collaboration with a third sector organisation was essential to ‘broker’ recruitment and the terms of this collaboration were clearly defined in the planning stages. In hindsight, we should have also created a collaboration agreement with our reference group members to help us all understand each other’s expectations. Certain timing and funding constraints meant that the pace of the research and the need to begin running the workshops, did not always match the availability of the reference group members. Overall working with the reference group enhanced every stage of the research process by adding alternative perspectives, exploring the feasibility and acceptability of questions and activities, challenging the researchers assumptions and forging collaborations and friendships that have lasted beyond the project ^27^.

### Strengths and limitations

This is the first study that we are aware of that directly engages with people with a learning disability about their views and experiences of anticipatory care planning. We collaborated with a learning disability advocacy organisation and established a Reference Group of people with lived experience of learning disability who were involved at all stages of the research. Thus, we are confident that our research captures the experiences and needs of people with a learning disability in participating in emergency care treatment planning. The requirement for the reference group to meet in person limited our recruitment sample and there were some initial challenges in recruiting and retaining reference group members which may have reduced its impact in the early stages of the study. Workshops were held on-line which again limited recruitment to those who could access and use electronic communications. Offering both focus group and individual interviews of carers of people with a learning disability increased accessibility for participation. However, the numbers of participants in all elements of the study are relatively low, and the views of participants may not capture the views of the wider learning disability community.

### Future research

Future research should include evaluation of the implementation and efficacy of the resources we have developed. Future implementation and training will need to take into account the wider findings of our study including the need for personalised support, presence of family members when appropriate, and avoidance of assumptions about capacity. In addition, the wider challenges of implementing change within an institution ^43^, and in particular the context of a general mistrust of health care services among people with a learning disability and their carers should be considered and mitigation strategies developed. 

## Conclusions

Emergency care planning and ReSPECT are viewed positively by people with a learning disability and family carers as a means to support self-determination of people with a learning disability, and to ensure the perspective of the patient is known in the absence of a family carer or advocate. However, there are significant doubts about the ability of the health system to ensure that the process will work effectively in practice to care well for people with a learning disability in an emergency. Previous negative experiences of the health care system have an impact on the level of trust family carers would place in emergency care treatment planning. To ensure emergency care treatment planning work well for people with a learning disability and their family carers, attention should be given to reasonable, personalised adjustments to support people with a learning disability to participate in planning conversations. There is a wider challenge of engendering increased trust in the health care system regarding treatment and care of people with a learning disability.

## Consent

Verbal or written informed consent was obtained from the participants and recorded on the study consent form. This is a reasonable adjustment to the consent process for people with limited literacy. Participants were offered the option to provide consent in writing or verbally. If verbal consent was given the participant name, the date and the name and role of the person taking consent would then be recorded on the consent form. This approach to consent was approved by London South East Research Ethics Committee (REC reference 21/LO/0455).

## Data Availability

We are unable to share underlying qualitative data in the form of transcripts due to ethical considerations. Full anonymised transcripts are not available. All data requests should be submitted to the PI Anne Slowther ( a-m.slowther@warwick.ac.uk) for consideration. Access to coding frameworks may be granted following review. No comments were made during ethical review about our open data sharing policies. University of Leeds: Extended data for ‘Autonomy & advocacy in planning for a medical emergency: Adults with a learning disability and family carers’ experiences and perceptions of the Recommended Summary Plan for Emergency Care and Treatment (ReSPECT) process’ is available from University of Leeds at https://doi.org/10.5518/1497 ^24^ Data are available under the terms of the Creative Commons Attribution 4.0 International license (CC-BY 4.0)
