# Supplementary material for: Autonomy & advocacy in planning for a medical emergency: Adults with a learning disability and family carers’ experiences and perceptions of the Recommended Summary Plan for Emergency Care and Treatment (ReSPECT) process
Source: NIHR Open Res. 2024 Oct 10;4:8. Originally published 2024 Mar 8. [Version 2] doi: 10.3310/nihropenres.13555.2 (PMC11496935; doi:10.3310/nihropenres.13555.2)
Supplement: Peer review history

# Reviewer report: Reviewer response for version 2

**DOI:** 10.3310/nihropenres.14855.r33140

**Published:** 2024-11-22

**Authors:** Ruth Northway

**Affiliations:** 1 University of South Wales, Pontypridd, Wales, UK

Thank you for revising and resubmitting this paper. Please note that I was not involved in the original review hence apologies if I am raising any new issues that were not highlighted in the original review.

 Overall the paper is well written and the study clearly addresses an important issue in the context of healthcare for people with a learning disability. It explores views and experiences in relation to ReSPECT from the perspectives of people with learning disabilities and family carers. This is achieved through a series of online workshops, focus groups and interviews. Data were thematically analysed. It is positive to note the use of inclusive research methods and the involvement of people with learning disabilities in a reference group. The findings are used to develop materials to support involvement of people with learning disabilities in engaging with the ReSPECT process. However, organisational challenges to effecting change within healthcare systems are also identified.

 I have commented 'partly' in relation to the criterion concerning availability of source data but acknowledge and support the explanation provided by the authors in the text.

 Generally the methods used are appropriately described but I do have a couple of queries. First in relation to data analysis it is made clear that analysis of the workshop data was both inductive and deductive. In relation to the focus groups and interviews, however, it is stated that data were initially coded using the interview guide (deductive). Were they then also analysed in an inductive manner? I also wonder whether development of a figure that shows data analysis of the two different sources and then  cross data set analysis to arrive at the final themes might be helpful for the reader.

 Also, in relation to the workshops, was there a particular reason that they were held online and what were the challenges (and possible benefits) of this approach? This information might be useful to other researchers.

 Finally, in relation to participant characteristics is it the case that the workshops primarily involved those with mild/ moderate learning disabilities whilst the family focus groups / interviews involved family members supporting those with more severe and profound learning disabilities? It would be helpful to the reader to have this information since the term 'learning disability' is used in relation to individuals with a wide range of strengths and support needs and the issues in relation to ReSPECT are likely to impact differently depending on the level and nature of impairment. 

 I also have a couple of other more general observations. First (and this is a minor point) but reference is made to CHANGE  before a description of the organisation is made in the Patient and Public Involvement section. This first reference may be confusing to a reader who is not familiar with the organisation. I would suggest either placing the explanatory material earlier or, after the first reference, inserting '(see below)'.

 My final point is perhaps best considered in relation to future research. In the current study 'carers' appears to include just family carers. However, some people with learning disabilities may have little or no contact with family and hence paid carers may be involved in providing support. Their position / relationship is different and is viewed differently by health professionals (see CIPOLD Report). It may therefore be worthy of exploration in future research.

 I hope these comments are helpful in further developing the paper.

Is the work clearly and accurately presented and does it cite the current literature?

Yes

If applicable, is the statistical analysis and its interpretation appropriate?

Not applicable

Are all the source data underlying the results available to ensure full reproducibility?

Partly

Is the study design appropriate and is the work technically sound?

Yes

Are the conclusions drawn adequately supported by the results?

Yes

Are sufficient details of methods and analysis provided to allow replication by others?

Partly

Reviewer Expertise:

Health needs of people with learning disabilities. Participatory / inclusive research

I confirm that I have read this submission and believe that I have an appropriate level of expertise to confirm that it is of an acceptable scientific standard.

# Reviewer report: Reviewer response for version 2

**DOI:** 10.3310/nihropenres.14855.r33139

**Published:** 2024-11-04

**Authors:** Angela Henderson

**Affiliations:** 1 University of Glasgow School of Health and Wellbeing, Glasgow, UK

This study investigates the use of Emergency Care Treatment Plans (ECTPs) for the population with learning disabilities, using an inclusive research methodology. This is an important and innovative study that reinforces the value of including the views and experiences of people with learning disabilities in health research. The study focuses on the Respect model of ECTPs. 

 The introduction effectively contextualizes and provides a robust rationale for the study, with references to existing relevant research on the health inequalities experienced by people with learning disabilities and the inequalities observed by other research in relation to emergency care treatment planning.

 The methods are clearly described - however it would be helpful to include a brief statement on the purpose and value of autoethnography. There are some minor grammatical and punctuation errors in the PPI section. Also the terms family-carers and relative-carers are used interchangeably - it would be preferable to select one of these for consistency.

 The results are well presented.

 The discussion integrates all of the themes to address the aims effectively within the context of other research. I wonder if the last paragraph before strengths and limitations would sit better under the strengths and limitations section?

Is the work clearly and accurately presented and does it cite the current literature?

Yes

If applicable, is the statistical analysis and its interpretation appropriate?

Not applicable

Are all the source data underlying the results available to ensure full reproducibility?

No

Is the study design appropriate and is the work technically sound?

Yes

Are the conclusions drawn adequately supported by the results?

Yes

Are sufficient details of methods and analysis provided to allow replication by others?

Yes

Reviewer Expertise:

Inclusive research, health and health care inequalities in population with intellectual (learning disabilities)

I confirm that I have read this submission and believe that I have an appropriate level of expertise to confirm that it is of an acceptable scientific standard.

# Reviewer report: Reviewer response for version 2

**DOI:** 10.3310/nihropenres.14855.r33094

**Published:** 2024-10-22

**Authors:** Irene Tuffrey-Wijne

**Affiliations:** 1 Kingston University, London, England, UK

I thank the authors for taking my comments into consideration. They have been satisfactorily addressed. I accept version of the paper.

Is the work clearly and accurately presented and does it cite the current literature?

Yes

If applicable, is the statistical analysis and its interpretation appropriate?

Not applicable

Are all the source data underlying the results available to ensure full reproducibility?

Yes

Is the study design appropriate and is the work technically sound?

Yes

Are the conclusions drawn adequately supported by the results?

Yes

Are sufficient details of methods and analysis provided to allow replication by others?

Partly

Reviewer Expertise:

NA

I confirm that I have read this submission and believe that I have an appropriate level of expertise to confirm that it is of an acceptable scientific standard.

# Reviewer report: Reviewer response for version 2

**DOI:** 10.3310/nihropenres.14855.r33093

**Published:** 2024-10-17

**Authors:** Rachael Cvejic

**Affiliations:** 1 Department of Developmental Disability Neuropsychiatry, School of Clinical Medicine, Faculty of Medicine & Health, UNSW Sydney, Sydney, New South Wales, Australia; 2 National Centre of Excellence in Intellectual Disability Health, Faculty of Medicine & Health, UNSW Sydney, Sydney, New South Wales, Australia

Thank you for the opportunity to review this revised manuscript. The authors have addressed all of my comments.

Is the work clearly and accurately presented and does it cite the current literature?

Yes

If applicable, is the statistical analysis and its interpretation appropriate?

Not applicable

Are all the source data underlying the results available to ensure full reproducibility?

Yes

Is the study design appropriate and is the work technically sound?

Yes

Are the conclusions drawn adequately supported by the results?

Yes

Are sufficient details of methods and analysis provided to allow replication by others?

Partly

Reviewer Expertise:

intellectual disability, inclusive research, mental health

I confirm that I have read this submission and believe that I have an appropriate level of expertise to confirm that it is of an acceptable scientific standard.

# Reviewer report: Reviewer response for version 1

**DOI:** 10.3310/nihropenres.14714.r31283

**Published:** 2024-04-22

**Authors:** Rachael Cvejic

**Affiliations:** 1 Department of Developmental Disability Neuropsychiatry, School of Clinical Medicine, Faculty of Medicine & Health, UNSW Sydney, Sydney, New South Wales, Australia; 2 National Centre of Excellence in Intellectual Disability Health, Faculty of Medicine & Health, UNSW Sydney, Sydney, New South Wales, Australia

Thank you for the opportunity to review this manuscript, which explores the views of people with intellectual disability and family carers about the Recommended Summary Plan for Emergency Care and Treatment (ReSPECT). This manuscript addresses an important area as people with intellectual disability have been historically excluded from discussions or processes related to future healthcare planning.

**Introduction:** The introduction is well written and summarises key issues. I would recommend providing some background information on inclusive research and its value, particularly in this area.

**Methods:** It would be helpful to see some further detail about the project reference group; the authors note that five members were initially recruited but that some members had to withdraw. It is not clear how many members withdrew and how many new members joined in the second round of recruitment, and at which point in the project lifecycle this occurred. It is also currently not clear whether reference group members are considered as research participants themselves, or as part of the research team. It would be helpful to have an initial outline of the methods (groups involved including number of people for each and project components) before expanding into the specifics of each. I also found the analysis section a bit unclear and would find it helpful to see some more information about the differences in analysis for the two formats as well as the process for combining them in the final analysis.

**Results: **The themes are articulated clearly and the supporting quotes are helpful.

**Discussion: **The discussion is concise and highlights the views of people with intellectual disability and family carers about the ReSPECT process, reasonable adjustments needed to support conversations, and issues that could impact implementation. I would be interested to see the addition of any recommendations from the authors perspectives that might help to address issues that could impact implementation (e.g., possible mechanisms to combat assumptions about capacity). It would also be relevant to note the importance of evaluating the resources including how the findings of this project could inform implementation and evaluation.

 Overall the manuscript is well written and makes a valuable contribution to this field. It is great to see the resources are linked and available for download.

Is the work clearly and accurately presented and does it cite the current literature?

Yes

If applicable, is the statistical analysis and its interpretation appropriate?

Not applicable

Are all the source data underlying the results available to ensure full reproducibility?

Yes

Is the study design appropriate and is the work technically sound?

Yes

Are the conclusions drawn adequately supported by the results?

Yes

Are sufficient details of methods and analysis provided to allow replication by others?

Partly

Reviewer Expertise:

intellectual disability, inclusive research, mental health

I confirm that I have read this submission and believe that I have an appropriate level of expertise to confirm that it is of an acceptable scientific standard, however I have significant reservations, as outlined above.

# Response

**Published:** 2024-08-06

**Authors:** Amy Russell

**Affiliations:** School of Medicine, University of Leeds, Leeds, England, UK

Thank you for your review and your kind words about the importance of the research. We have made the following changes in response to your review:

  

1. We have now included a section in the **Introduction** on the value of inclusive research in this area and situating our study within the literature 
2. In response to the confusion about the Reference Group we have now added an **Overview of the Study** at the beginning of the methods section to clarify each element and the role of the reference group. We hope this clarifies the role of the reference group in both our study overview and our expanded PPI section, explaining their dual involvement as advisors on the research process and outcomes, and as participants in an autoethnographic analysis of their involvement in the study.
3. In **Methods** we have added detail about the number of reference group members who withdrew and the timing of replacements in our expanded section on patient and public involvement ( **PPI**).
4. In **Methods** we have now expanded our analysis section to clarify both the individual analyses for focus groups and workshops and how these were combined for this paper.
5. We added a section called **Future Research** where we noted the importance of evaluating implementation of the resources as future research including the importance of taking into account our wider study findings on the context in which resources are implemented.

# Reviewer report: Reviewer response for version 1

**DOI:** 10.3310/nihropenres.14714.r31282

**Published:** 2024-03-29

**Authors:** Irene Tuffrey-Wijne

**Affiliations:** 1 Kingston University, London, England, UK

I would like to congratulate the authors on conducting a co-produced study that included people with a learning disability in a meaningful way, on a topic that affects them but from which they have historically been excluded.

 This article describes a qualitative study exploring the views of people with a learning disability and family carers with regards to ReSPECT, using online interactive workshops with people with a learning disability and focus group/interviews with family carers. The study was carried out by a team of researchers working with a Reference Group of people with a learning disability. The findings show that whilst both participant groups were supportive of ReSPECT planning (leading to study outputs that includes an easy-read guide to support ReSPECT planning with people with a learning disability), they were dubious about the ability of the healthcare system to incorporate this.

 I have a number of suggestions for changes or additions that will improve the article, in particular with regards to clarity around the methods.

**ABSTRACT: Methods: **I think it is important and relevant to mention the inclusive methodology here, involving a Reference Group of people with a learning disability. I would also like to see numbers of participants added.

**INTRODUCTION: **This clearly sets out the issues, and broadly sets out the current state of the art, although some of the literature cited is quite old [Ref 1]. As the study is concerned with a UK-based healthcare system and planning process, it may also help (if the article work limit allows) to specify where cited research originates (for example, Wagemans’ study is 14 years old and conducted in the Netherlands, where health/social care systems are different). It may be worth adding a small section to the introduction that sets out the innovative nature of inclusive research in this area. For example, is the methodological choice of working with a Reference Group based on precedents in the literature? How about conducting workshops online? Or did the researchers enter unchartered territory? Alternatively, this could be an added section in the discussion.  

**METHODS: **The chosen methodology is appropriate and innovative. However, the article would benefit from further clarity about the different groups involved, particularly with regards to the Reference Group. I was a bit confused whether this group was seen as part of the research team (as they were involved in developing part of the methodology, and are also described as being involved in data analysis and production of outputs) or whether they were “participants” (which is how they are described, including a participant recruitment process). How many Reference Group members were there? (the article mentions 5 initial members followed by further recruitment). Were they paid for their time? If they were participants, what was the difference with the workshop participants? (I note that one workshop participant also helped with analysis, adding to the confusion). Did the two CHANGE members who worked with the study team to develop the study design have a learning disability? Perhaps the easiest and clearest way of clarifying this is by starting the methods section with a clear and brief overview of what you did (including the workshops and focus groups/interviews, as described in the abstract; but also the Reference Group, as that was part of the methodology), before expanding. Currently, the focus groups are not mentioned until “recruitment”, and their purpose doesn’t become clear until the end of the “Workshop development” section, where it doesn’t seem to belong as it’s not part of the workshops). It may help to produce a figure or table showing everyone involved: research team, reference group, workshop participants, focus group/interview participants.

 DATA ANALYSIS: I am not sure I understand what is meant by code (b): Reflecting the meaning of emergency care treatment planning for people with a learning disability? Perhaps a supplementary file with some examples (e.g. extracts of data and related codes) might help.

 It is wonderful to see that people with a learning disability were involved in data analysis, but it would be helpful to have some indication of the actual contribution they made to the analysis. Did the feedback to the findings they provided change the analysis and subsequent outputs? Some examples might be helpful.

**RESULTS:** This section is clear.

**DISCUSSION: **The discussion describes both the reasonable adjustments needed to make the ReSPECT process accessible (leading the production of new easy-read resources by the study team), and the rather huge barrier of ensuring that health/social care staff actually use them. This is clearly a deep-rooted issue, echoed in other studies and reports. This will undoubtedly have an effect on the implementation (or not) of the newly developed materials. It would be important to make some reference to this, including the need to evaluate the implementation and effectiveness of the new resource, or any other future work/studies needed to ensure people with a learning disability are as involved in the ReSPECT process as they want to be.

 If space allows, it would also be valuable to see some reflection on the inclusive methodology, and any lessons learnt.

 Reference 28 is quite old (2007) and there has been more recent work around older carers – for example,

 Ref [1].

 I hope these comments are helpful. This is an extremely worthwhile study, and I’m glad to see it published.

Is the work clearly and accurately presented and does it cite the current literature?

Yes

If applicable, is the statistical analysis and its interpretation appropriate?

Not applicable

Are all the source data underlying the results available to ensure full reproducibility?

Yes

Is the study design appropriate and is the work technically sound?

Yes

Are the conclusions drawn adequately supported by the results?

Yes

Are sufficient details of methods and analysis provided to allow replication by others?

Partly

Reviewer Expertise:

Learning disability, palliative care, inclusive research, qualitative research

I confirm that I have read this submission and believe that I have an appropriate level of expertise to confirm that it is of an acceptable scientific standard, however I have significant reservations, as outlined above.

## References

[1] : 'There's a timebomb': Planning for parental death and transitions in care for older people with intellectual disabilities and their families. *J Appl Res Intellect Disabil* .2024;37(2) : 10.1111/jar.13174 e13174 10.1111/jar.13174 38093532

# Response

**Published:** 2024-08-06

**Authors:** Amy Russell

**Affiliations:** School of Medicine, University of Leeds, Leeds, England, UK

Thank you for your review and your kind words about the research. We have made the following changes in response to your review:

  

1. We have updated the **abstract** to contain participant numbers.
2. In the **Introduction** we have stated that previous research comes from a variety of different health care systems but that UK studies are included, thus indicating that findings reflect experience in UK health care.
3. We have added a section to the **Introduction** describing the importance of inclusive research in this area and situating our research in this emerging field. We have also included a section in the discussion on our reflections on the inclusive research in the project. 
4. In response to the confusion about the Reference Group we have now added an **Overview of the Study** at the beginning of the methods section to clarify each element and the role of the reference group. 
5. In **Methods** we have clarified the description of our analysis process which included deductive coding for issues relating to information, process, and support for engaging with the ReSPECT process (which fed into development of resources), and inductive coding to capture participants views on the meaning of emery care planning for people with a learning disability.
6. In the **PPI section** we expanded on the contribution of the reference group to the analysis.
7. We have now added a section to our **Discussion **reflecting on the inclusive methodology, challenges and lessons learned.
8. We added a section called **Future Research** where we note the importance of evaluating implementation of our resources and the importance of taking into account our wider study findings around involving people with a learning disability in the ReSPECT process, including the wider mistrust of NHS systems in providing care for people with a learning disability.
9. We have updated our **References**, thank you for the suggestion.
